# Revision Endoscopic Proximal Hamstring Repair with Suture Staples

**DOI:** 10.1016/j.eats.2025.103928

**Published:** 2025-10-07

**Authors:** Christian L. Blough, Michael B. Banffy

**Affiliations:** Cedars-Sinai Department of Orthopaedic Surgery, Division of Sports Medicine, Los Angeles, California, U.S.A.

## Abstract

Proximal hamstring tears can significantly affect patient quality of life. Recently, endoscopy has been used to address these tears when conservative management fails. The results of endoscopic proximal hamstring repair are promising, but retears do occur. Revision endoscopic repair is an option. We present a technique, using suture staples, which can be used in partial or undersurface proximal hamstring tears.

Proximal hamstring injuries are debilitating injuries that most commonly affect athletic or active individuals and can result in significant weakness, pain, and functional limitations if they fail conservative management.^1^ While open surgical repair has traditionally been the standard for surgical management, endoscopic techniques have gained popularity in recent years due to decreased morbidity, superior cosmesis, and comparable clinical outcomes.^2^, ^3^, ^4^ However, revision proximal hamstring repair remains technically challenging, particularly in the setting of scarring, altered anatomy, and compromised tendon quality.

Achieving secure tendon-to-bone fixation is paramount. The senior author (M.B.B.) has seen multiple instances of patient’s reporting snapping symptoms with hip flexion and extension in the setting of undersurface tears of the proximal hamstring tendon. We hypothesize that this is from the proximal tendon insertion being intact and acting as a pivot point for the remaining tendon. As the hip is flexed and the hamstring tendon becomes taught, the distal aspect of the proximal hamstring tendon can snap over the ischial tuberosity. In this setting, traditional suture anchor constructs may not provide sufficient fixation strength or footprint compression in scarred or attenuated tissues, increasing the risk of repair failure. To address these challenges, novel fixation constructs have been explored endoscopically in other areas of the hip. Hartigan et al. described the use of suture staples for undersurface abductor tendon repair, which can provide broad-based compression across the tendon–bone interface and improve load distribution.^5^ Additionally, the use of bioinductive implants as an augment has been shown to be beneficial in rotator cuff tear repair and have previously been described for proximal hamstring tear repair.^6^

The purpose of this article is to describe a technique for revision repair of a partial, undersurface tear of the proximal hamstring tendon using suture staples and patch augmentation.

## Surgical Technique

**Fig. 1:**
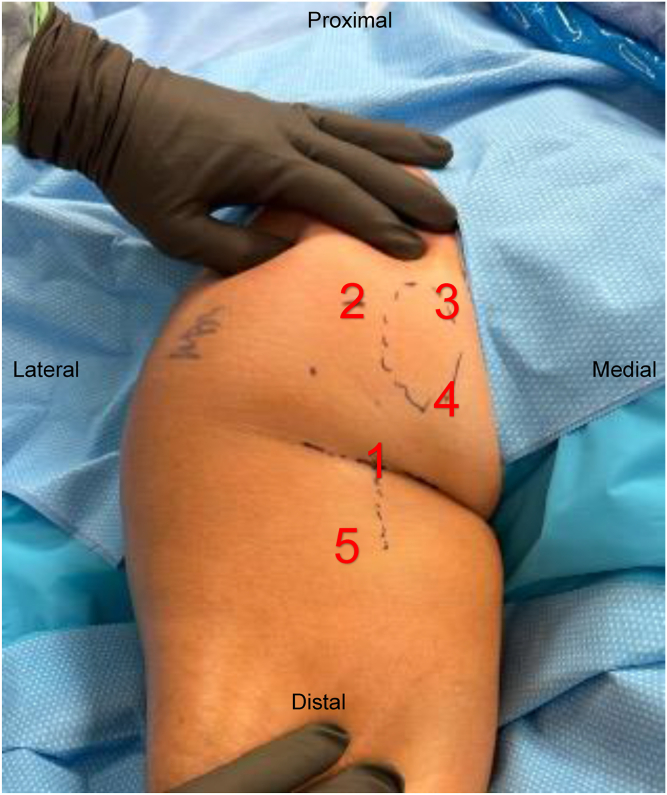
Exterior view of the left buttock and proximal thigh with the patient positioned prone. (1) Direct posterior portal. (2) Superolateral portal. (3) Percutaneous superomedial portal. (4) Percutaneous medial portal. (5) Distal accessory portal.

**Fig. 2:**
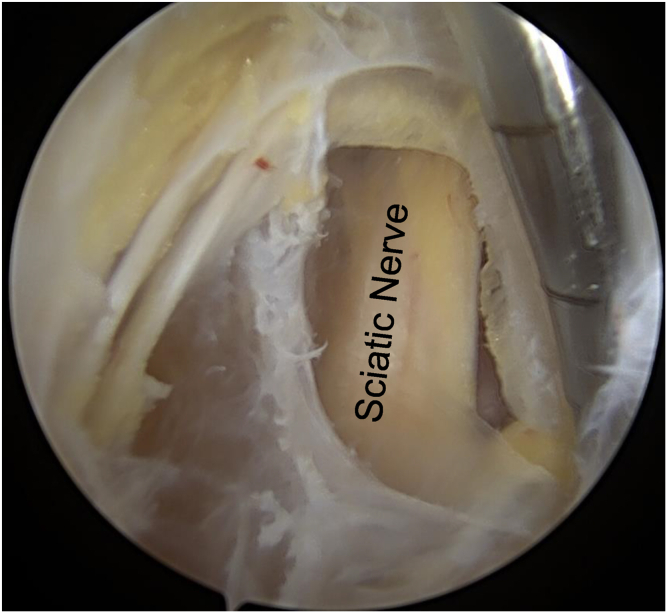
Left hip endoscopy viewing from the posterior portal. The sciatic nerve is visible post-neurolysis. The shaver is coming from the superolateral portal.

The patient is positioned prone on a well-padded table under general anesthesia. A direct posterior portal is created in the gluteal crease, centered over the ischial tuberosity, labeled 1 in Fig 1. A 30° arthroscope is introduced into the space between the gluteus maximus and ischial tuberosity. A superolateral portal, labeled 2 in Fig 1, is then created a few centimeters lateral to the proximal ischial tuberosity. A shaver and radiofrequency ablator are then used through the superolateral portal to remove scar tissue and perform an ischial bursectomy. Careful blunt dissection using the back of the shaver is then performed lateral to the ischial tuberosity to locate the posterior femoral cutaneous and sciatic nerves. Locating these nerves is paramount to safely performing endoscopic hamstring repair, especially in the revision setting. Neurolysis of both nerves is then performed. The appearance of the sciatic nerve postneurolysis can be seen in Fig 2.

**Fig. 3:**
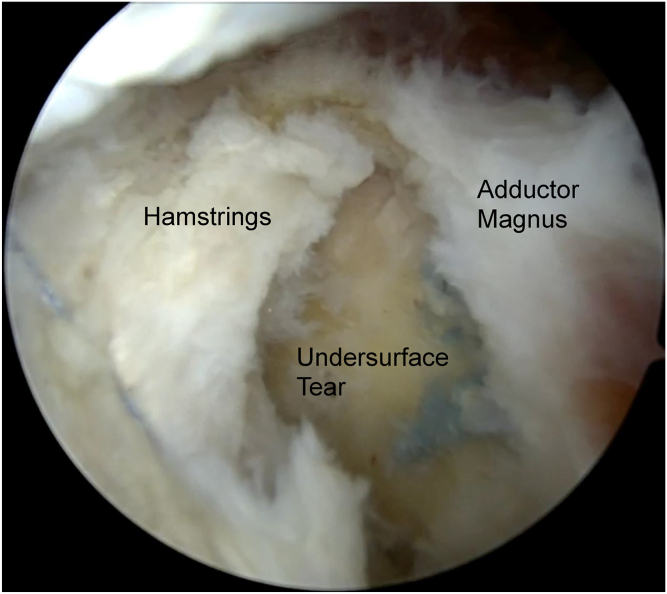
Left hip endoscopy viewing from the posterior portal. The large undersurface tear is noted after the interval between the hamstring and adductor magnus is opened.

The prior proximal hamstring repair is then assessed. Visually, the repair may appear intact, with the hamstring in an anatomic position but with the adductor magnus slightly posterior. Probing may reveal an unstable adductor magnus tendon, as well as an unstable distal hamstring footprint. The interval between the adductor magnus and hamstring is created as seen in Fig 3. This allows for visualization of the lateral footprint. Further probing is then performed to assess for undersurface tears.

**Fig. 4:**
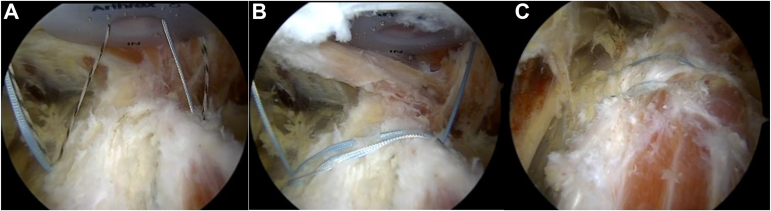
Left hip endoscopy viewing through the direct posterior portal. (A) 1.8-mm Q-Fix all-suture knotless anchors have been placed at the proximal lateral and medial ischial tuberosity. (B) The anchors have been coupled to create a suture staple. (C) The suture staples have been tightened, creating tendon-bone compression over the ischial tuberosity.

A suture is passed through the hamstring tendon to allow for easier manipulation and visualization of the footprint. A high-speed burr (5.0-mm burr, Arthrex, Naples, FL) is used to clear the footprint to a bleeding bony bed. Remaining suture from the prior repair is then removed. A 1.8 mm all-suture knotless anchor (Q-Fix, Smith & Nephew, Watford, UK) is then placed proximally in the hamstring tendon on the lateral ischial tuberosity through the posterolateral portal. A superomedial percutaneous portal, labeled 3 in Fig 1, is then created under direct visualization with a spinal needle directly over the ischial tuberosity, a few centimeters medial to the superolateral portal. This portal allows for better trajectory to the medial ischial tuberosity. A second 1.8-mm all-suture knotless anchor (Q-Fix, Smith & Nephew) is then placed proximally in the adductor magnus tendon on the medial ischial tuberosity through this superomedial portal. All sutures are then retrieved through the superolateral portal. The repair suture from the lateral anchor is passed through the shuttle suture from the medial anchor and vice versa. The repair sutures are then simultaneously tightened, providing broad compression of the tendon between the anchors to the ischial tuberosity in a staple-like fashion. The suture ends are then cut. This can be visualized in Fig 4.

This technique is repeated in a similar fashion more distally to provide compression over the distal ischial tuberosity. A second percutaneous medial portal, labeled 4 in Fig 1, is made over the distal medial aspect of the ischial tuberosity to allow for the distal medial anchor to be placed. After tightening, the repair is then probed to assess for stability of the tendons on the ischial tuberosity.

**Fig. 5:**
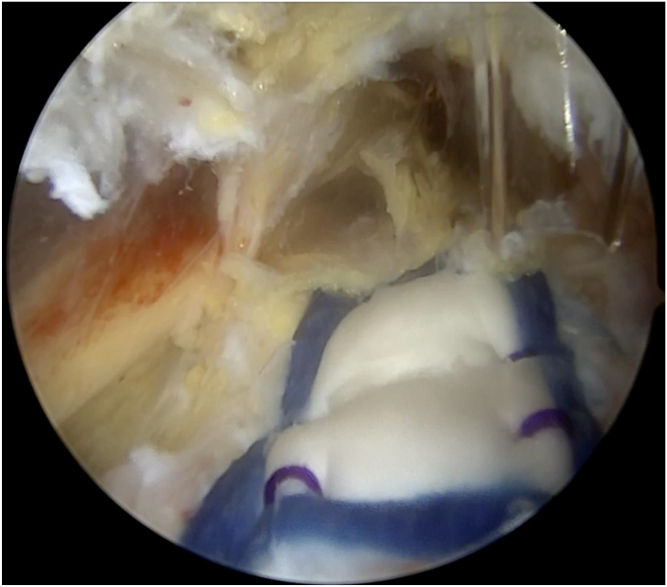
Left hip endoscopy viewing from the posterior portal. The bioinductive patch has been placed over the repair in line with the hamstring fibers and secured in place.

A distal accessory portal, distal to the gluteal crease and slightly lateral, labeled 5 in Fig 1, is then made under direct visualization with a spinal needle to allow placement of the bioinductive patch (Regeneten, Smith & Nephew) over the repair in a parallel orientation to the hamstring tendon. The bioinductive patch is passed through this accessory portal and deployed over the center of the ischial tuberosity. Staples are placed around the periphery of the patch to secure it to the tendon. This can be seen in Fig 5. The inserter is removed. Skin closure is performed. This technique is demonstrated in Video 1 and summarized in Table 1.

**Table 1:** Surgical Steps of the Proximal Hamstring Suture Staple Repair

Surgical Steps	
1	Diagnostic endoscopy, ischial bursectomy, and neurolysis
2	Assess hamstring origin for stability and tears.
3	Remove prior suture and implants.
4	Prepare footprint.
5	Place proximal row anchors, link anchors, and tighten, creating suture staple.
6	Place distal row anchors, link anchors, and tighten, creating suture staple.
7	Assess stability of repair.
8	Create accessory distal portal for patch placement.
9	Pass patch, deploy, and secure with biodegradable staples.
10	Assess stability of patch, remove instruments, and perform closure.

### Postoperative Care

The patient is placed on naproxen 500 mg twice daily for the first 3 weeks postoperatively for heterotopic ossification prophylaxis. The patient can perform protected weight bearing with crutches for the first 6 weeks, followed by unrestricted weight bearing, stretching, and closed chain exercised for the next 6 weeks. At 3 months, there is no limitation on the range of motion and gradual strengthening exercises are permitted.

## Discussion

Proximal hamstring injuries can be debilitating injuries, especially for active patients. Endoscopic surgical repair has recently been advocated for these active patients to restore their function. This approach is not without complications, and chronic pain and retear are possible. Revision surgery for retear or recurrence of symptoms has been reported to be >10%.^7^ Further, revision surgery can be technically complex, and evidence guiding treatment is limited.

Open surgical repair is an option, with the theoretical advantage of wider exposure and more fixation options in the revision setting. There have not yet been any studies showing a difference in outcomes between open and endoscopic techniques.^7^^,^^8^ Given the advantages of decreased morbidity, lower postoperative pain, and improved cosmesis, endoscopic revision is an attractive option.

The suture staple construct described in this technique provides broad tendon-to-bone compression with minimal suture burden. This is particularly useful in undersurface tears where the proximal origin is intact, but the distal origin is mobile. In revision settings, preserving native biology is essential to optimize healing, and this construct avoids disrupting the remaining, intact footprint. Additionally, the knotless mechanism reduces surgical time and knot prominence, which could potentially lead to soft tissue irritation. Finally, linking of the anchors together shares the load between anchors, increasing the strength of the construct. Strengths and weaknesses of this technique can be found in Table 2.

**Table 2:** Pearls and Pitfalls of the Proximal Hamstring Suture Staple Repair

Pearls	Pitfalls
Perform extensive ischial bursectomy and neurolysis for safe visualization	Most useful in partial, undersurface tears
Provides broad compression of tendon-bone interface	Patch augmentation may be costly
Minimal disruption to existing biology	Creation of holes in hamstring/adductor magnus tendon for anchor insertion
No knot tying required	

Bioinductive collagen patches have been shown to enhance healing in large and massive rotator cuff tears by increasing tendon vascularity and thickness.^9^ These factors are thought to decrease strain at the repair site as well, which can lower retear rates.^10^ A recent systematic review of randomized control trials by Orozco et al. revealed lower retear rates with the use of patch augmentation in large rotator cuff tears.^11^ Although data are limited for its use in proximal hamstring repair, it has been previously described.^6^ Given the favorable safety profile, we advocate its use as an adjunct in select revision cases.

This technique demonstrates a reproducible, minimally invasive method for revision proximal hamstring repair with a suture staple construct and bioinductive path augmentation. It provides broad tendon compression, preserves the intact footprint, and includes augmentation to improve healing.

## Disclosures

The authors declare the following financial interests/personal relationships which may be considered as potential competing interests: M.B.B. reports consulting and advisory fees from 10.13039/100009026Smith & Nephew and Vericel Corporation, and he holds a patent with royalties. The other author (C.L.B.) declares that they have no known competing financial interests or personal relationships that could have appeared to influence the work reported in this paper.

## Supplementary Data

Video 1 This video depicts a left revision proximal hamstring repair using a suture staple construct with bioinductive patch augmentation. The patient is positioned prone on a regular table. A direct posterior portal is created and used at the primary viewing portal. A superolateral portal is created and used as the primary working portal. An extensive ischial bursectomy is performed. Neurolysis of the posterior femoral cutaneous nerve and sciatic nerve is next performed. The hamstring and adductor magnus origin are then investigated and an interval between the two is created. The prior sutures are then removed, and preparation of the ischial footprint is performed with a burr. The 1.8-mm Q-Fix knotless all-suture anchors are then placed on the lateral and medial aspects of the proximal ischial tuberosity. The anchors are then coupled and tightened to provide compression across the tendon origin. This is again repeated distally. The Regeneten bioinductive patch is then placed through a distal accessory portal and secured with bioabsorbable staples.

## References

[1] Cohen S., Bradley J. (2007). Acute proximal hamstring rupture. J Am Acad Orthop Surg.

[2] Belk J.W., Kraeutler M.J., Mei-Dan O., Houck D.A., McCarty E.C., Mulcahey M.K. (2019). Return to sport after proximal hamstring tendon repair: A systematic review. Orthop J Sports Med.

[3] Bodendorfer B.M., Curley A.J., Kotler J.A. (2018). Outcomes after operative and nonoperative treatment of proximal hamstring avulsions: A systematic review and meta-analysis. Am J Sports Med.

[4] Fenn T.W., Timmermann A.P., Brusalis C.M., Kaplan D.J., Ebersole J.W., Nho S.J. (2023). Clinical outcomes after open and endoscopic repair of proximal hamstring tendon tears at a minimum follow-up of 5 years. Orthop J Sports Med.

[5] Hartigan D.E., Mansor Y., Perets I., Walsh J.P., Mohr M.R., Domb B.G. (2018). Knotless “Suture Staple” technique for endoscopic partial thickness abductor tendon repair. Arthrosc Tech.

[6] Hamula M.J., Cady A., Yousefzadeh K., Banffy M. (2021). Endoscopic implantation of bioinductive patch for chronic partial retearing after hamstring repair. Arthrosc Tech.

[7] Ebert J.R., Edwards P.K., Ammann E. (2025). Older age and a non-sporting injury mechanism are associated with re-injury and the need for revision surgery over a minimum 2-year follow-up following proximal hamstring tendon repair. Knee Surg Sports Traumatol Arthrosc.

[8] Bowman E.N., Marshall N.E., Gerhardt M.B., Banffy M.B. (2019). Predictors of clinical outcomes after proximal hamstring repair. Orthop J Sports Med.

[9] Thon S.G., O’Malley L., O’Brien M.J., Savoie F.H. (2019). Evaluation of healing rates and safety with a bioinductive collagen patch for large and massive rotator cuff tears: 2-year safety and clinical outcomes. Am J Sports Med.

[10] Bushnell B.D., Bishai S.K., Krupp R.J. (2021). Treatment of partial-thickness rotator cuff tears with a resorbable bioinductive bovine collagen implant: 1-year results from a prospective multicenter registry. Orthop J Sports Med.

[11] Orozco E., Dhillon J., Keeter C., Brown T.D., Kraeutler M.J. (2024). Rotator cuff repair with patch augmentation is associated with lower retear rates for large tears: A systematic review of randomized controlled trials. Arthroscopy.

